# Impact of DPP-4 Inhibitors in Patients with Diabetes Mellitus and Heart Failure: An In-Depth Review

**DOI:** 10.3390/medicina60121986

**Published:** 2024-12-02

**Authors:** Francisco Epelde

**Affiliations:** Medicine Department, Parc Taulí Hospital Universitari, Institut d’Investigació i Innovació Parc Taulí I3PT, 08208 Sabadell, Spain; fepelde@gmail.com

**Keywords:** heart failure, diabetes mellitus, DDP-4 inhibitors

## Abstract

The increasing prevalence of both type 2 diabetes mellitus and heart failure has underscored the urgent need for optimized therapeutic strategies that address the complex interplay between these conditions. Dipeptidyl peptidase-4 (DPP-4) inhibitors have emerged as a popular class of glucose-lowering agents due to their favorable glycemic effects, safety profile, and potential cardiovascular benefits. However, the impact of DPP-4 inhibitors on heart failure outcomes in patients with diabetes remains contentious, with conflicting evidence from clinical trials and observational studies. This review critically examines current evidence on the use of DPP-4 inhibitors in patients with coexisting diabetes and heart failure, focusing on pharmacodynamics, safety, and efficacy outcomes. We explore the physiological mechanisms by which DPP-4 inhibitors may influence heart failure risk, including modulation of inflammation, oxidative stress, and myocardial fibrosis. Clinical trials such as SAVOR-TIMI 53, EXAMINE, and TECOS are evaluated to provide a comprehensive analysis of DPP-4 inhibitors’ effects on hospitalization for heart failure, mortality, and cardiovascular events in diabetic patients. While some trials suggest an increased risk of HF hospitalizations with specific DPP-4 inhibitors (e.g., saxagliptin), others report neutral effects, raising questions about the class effects versus individual drug characteristics within this group. Additionally, we address discrepancies in outcomes related to patient demographics, HF phenotype, and comorbid conditions that may influence DPP-4 inhibitors’ risk–benefit profile. Comparative insights into alternative glucose-lowering therapies such as SGLT2 inhibitors and GLP-1 receptor agonists are also provided, highlighting potential implications for treatment selection in this high-risk population. In summary, this review synthesizes available evidence on DPP-4 inhibitors’ impact in diabetic patients with heart failure, aiming to guide clinicians in making informed therapeutic decisions. While DPP-4 inhibitors remain a viable option in diabetes management, caution is warranted in patients with advanced heart failure, and future research is essential to refine patient-specific guidelines.

## 1. Introduction

Diabetes mellitus and heart failure are two highly prevalent, chronic, and interconnected diseases that impose significant burdens on health care systems worldwide. Diabetes is a major risk factor for the development of heart failure, and approximately 30–40% of patients with type 2 diabetes mellitus (T2DM) eventually develop heart failure. The overlap of these diseases not only complicates treatment but also exacerbates morbidity and mortality. For patients with both diabetes and heart failure, selecting antidiabetic therapies that address blood glucose levels without worsening cardiac function is essential [1].

Dipeptidyl peptidase-4 (DPP-4) inhibitors have emerged as an essential class of glucose-lowering drugs in managing diabetes, given their favorable profiles in terms of weight neutrality, low hypoglycemia risk, and, for many drugs in this class, ease of use in patients with varying renal function. However, their application in heart failure remains a topic of investigation due to concerns raised by clinical studies on specific agents [2]. This review aims to delve into the physiological underpinnings of DPP-4 inhibitors, analyze their clinical efficacy and safety for heart failure patients, explore the unique considerations for special populations, and compare them to other antidiabetic therapies in this high-risk group. This exploration will help in understanding the current positioning of DPP-4 inhibitors and will provide insights into future directions for research [3].

## 2. Physiopathology of Diabetes, Heart Failure, and DPP-4 Inhibitors

Diabetes and heart failure are both systemic diseases that share overlapping pathophysiological pathways. In diabetes, chronic hyperglycemia induces a multitude of cellular changes, leading to oxidative stress, inflammation, and endothelial dysfunction, all of which contribute to the development of both microvascular and macrovascular complications. These complications in turn precipitate cardiovascular diseases, including ischemic heart disease and heart failure [4].

Heart failure is characterized by the heart’s inability to meet the metabolic demands of the body, resulting in symptoms of fatigue, dyspnea, and fluid retention. In the context of diabetes, the myocardial structure and function undergo various alterations such as increased left ventricular mass, myocardial fibrosis, and impaired relaxation of the heart muscle, collectively known as diabetic cardiomyopathy [5,6]. The interplay between diabetes and heart failure creates a vicious cycle in which metabolic disturbances promote cardiac dysfunction, while cardiac dysfunction exacerbates insulin resistance and worsens glycemic control [7].

The DPP-4 enzyme, which is widely expressed in endothelial cells, the immune system, and other tissues, plays a central role in glucose metabolism by inactivating incretin hormones, including glucagon-like peptide-1 (GLP-1) and glucose-dependent insulinotropic polypeptide (GIP). Inhibition of DPP-4 extends the half-life of GLP-1, which increases insulin secretion and decreases glucagon release in a glucose-dependent manner. In addition to their effects on glucose regulation, GLP-1 and GIP have cardiovascular actions, including improvements in endothelial function, reduction in oxidative stress, and anti-inflammatory effects, which are potentially advantageous in heart failure management [8,9].

In patients with heart failure, DPP-4 expression and activity can be affected, and studies suggest a potential overexpression of the enzyme in the cardiac and endothelial tissues in heart failure conditions [10]. This upregulation of DPP-4 has critical implications for heart failure pathophysiology. When DPP-4 activity is increased, the degradation of GLP-1 is accelerated, leading to reduced levels of active GLP-1 in the circulation. GLP-1 plays an important cardioprotective role, including improving myocardial function, reducing oxidative stress, and enhancing endothelial function [11], which are essential in the setting of heart failure. GLP-1 receptor (GLP-1R) activation has shown benefits in terms of increasing myocardial glucose uptake, enhancing cardiac output, and providing anti-inflammatory effects, which could otherwise be diminished by DPP-4 overactivity [12].

The increased expression of DPP-4 in heart failure may therefore contribute to worsened outcomes by blunting these beneficial GLP-1-mediated effects. By inhibiting DPP-4, DPP-4 inhibitors prolong the activity of GLP-1, enhancing its cardioprotective properties, which could theoretically benefit patients with heart failure [13,14]. However, the relationship between DPP-4 inhibition and heart failure outcomes remains complex and has shown variable results in clinical trials. For instance, the SAVOR-TIMI 53 trial [15] raised concerns when it observed an increased risk of heart failure hospitalizations associated with the DPP-4 inhibitor saxagliptin, though other studies with different DPP-4 inhibitors, like sitagliptin, found no such association.

The reason for these differing results could be due to unique off-target effects of specific DPP-4 inhibitors or variations in their binding and inhibition of DPP-4 on different cell types, including those involved in cardiac function. Additionally, beyond GLP-1, DPP-4 also acts on other substrates that may influence immune responses and fibrotic processes in the myocardium [16], adding complexity to its role in heart failure. By inhibiting DPP-4, there is potential modulation of these additional pathways, which could variably impact heart failure depending on the patient’s underlying disease state and the specific DPP-4 inhibitor used [17].

Ultimately, while DPP-4 inhibitors offer a promising avenue to augment GLP-1 levels and support metabolic control in patients with heart failure, the potential overexpression of DPP-4 and its extensive influence on GLP-1 and other bioactive peptides suggest that the net effects on cardiac health are multifactorial. Future research aimed at understanding the exact role of DPP-4 expression in heart failure and differentiating the effects of specific DPP-4 inhibitors could help clarify their role in this patient population and optimize their safe application [18,19].

## 3. Mechanisms of Action of DPP-4 Inhibitors and Their Implications in Heart Failure

DPP-4 inhibitors, commonly referred to as gliptins, lower blood glucose by inhibiting the DPP-4 enzyme, thereby increasing the levels of active GLP-1 and GIP. These incretin hormones play essential roles in maintaining glucose homeostasis by enhancing insulin secretion and suppressing glucagon release. GLP-1 also slows gastric emptying, which helps to moderate postprandial blood glucose spikes, and has peripheral effects that improve glucose uptake in muscle tissues.

Beyond glucose regulation, GLP-1 has shown cardioprotective properties in preclinical and clinical studies. In animal models, GLP-1 has been observed to improve myocardial contractility [20], reduce myocardial injury after ischemic events, and enhance endothelial function through increased nitric oxide availability, leading to vasodilation and improved blood flow [21]. When GLP-1 binds to its receptor on cardiomyocytes, it initiates a cascade of intracellular signaling pathways that promote an increase in cyclic adenosine monophosphate (cAMP). cAMP serves as a secondary messenger that activates protein kinase A (PKA), which in turn phosphorylates various proteins involved in calcium handling within the heart cells. Calcium plays a central role in myocardial contractility, as it facilitates the interaction between actin and myosin—the contractile proteins in muscle cells. By enhancing calcium influx and mobilization, GLP-1 leads to improved myocardial contractility, increasing the strength and efficiency of heart contractions without significantly raising heart rate [22].

In addition to directly influencing calcium handling, GLP-1 also modulates myocardial metabolism, providing an energy substrate shift from fatty acids to glucose. This shift is particularly beneficial in conditions where the heart muscle is under stress, such as ischemia or heart failure, as glucose is a more oxygen-efficient fuel source than fatty acids. This metabolic adaptation reduces oxygen demand, thereby preserving cardiac function during periods of low oxygen supply [23]. This shift contributes to myocardial protection and enhanced contractility, especially in the setting of ischemic injury, where oxygen efficiency is crucial for cell survival and function.

Moreover, GLP-1 has been found to have positive effects on endothelial function and blood flow, indirectly benefiting myocardial contractility. By promoting vasodilation through the release of nitric oxide (NO) from endothelial cells, GLP-1 helps increase coronary blood flow, ensuring an adequate supply of oxygen and nutrients to the heart muscle. Improved blood flow supports optimal myocardial performance and can enhance the contractile response of the heart during periods of increased demand [24].

Experimental and clinical studies have demonstrated the benefits of GLP-1 in heart failure patients, where it has been associated with improved left ventricular ejection fraction (LVEF), a key measure of contractility. For example, in heart failure with reduced ejection fraction (HFrEF), GLP-1 infusion or therapy with GLP-1 analogs has shown improvements in LVEF, potentially due to a combination of enhanced calcium dynamics, metabolic efficiency, and better coronary blood flow [25]. These effects suggest that GLP-1 could be a therapeutic option in heart failure patients, providing both metabolic and contractile benefits [25,26].

Despite these promising findings, the exact mechanisms by which GLP-1 influences myocardial contractility are not entirely understood and likely involve complex interactions between metabolic, hemodynamic, and direct cardiomyocyte signaling pathways. Ongoing research aims to elucidate these pathways further, with the hope of leveraging GLP-1 and its analogs to enhance cardiac function in patients with compromised myocardial contractility. In summary, GLP-1 contributes to myocardial contractility through enhanced calcium handling, metabolic modulation, and improved coronary blood flow, making it a valuable therapeutic target in cardiac dysfunction, especially heart failure [27,28].

Moreover, GLP-1 reduces the production of pro-inflammatory cytokines, which may mitigate the chronic inflammation observed in heart failure and diabetes [29]. Glucagon-like peptide-1 (GLP-1) is an incretin hormone primarily known for its role in glucose regulation through the stimulation of insulin secretion and suppression of glucagon release. Beyond its effects on glucose homeostasis, GLP-1 has shown significant influence on inflammatory pathways, particularly through its interaction with cytokines. Cytokines are small signaling proteins that play a central role in the immune response, regulating inflammation, cell proliferation, and tissue repair [30]. This interaction between GLP-1 and cytokine regulation has drawn interest due to its potential therapeutic implications for chronic inflammatory conditions, including diabetes, obesity, cardiovascular diseases, and even neurodegenerative disorders.

GLP-1 and its analogs have been shown to exhibit anti-inflammatory properties, partly by modulating pro-inflammatory cytokines such as tumor necrosis factor-alpha (TNF-α), interleukin-6 (IL-6), and interleukin-1 beta (IL-1β). These cytokines are commonly elevated in metabolic diseases and contribute to the chronic low-grade inflammation associated with insulin resistance and cardiovascular complications. Studies indicate that GLP-1 reduces levels of these pro-inflammatory cytokines, which may help alleviate inflammation and protect against tissue damage in the pancreas, vasculature, and other organs affected by metabolic disorders [31].

The anti-inflammatory effects of GLP-1 are thought to be mediated through several mechanisms, including the activation of the GLP-1 receptor (GLP-1R) on immune cells such as macrophages. When GLP-1 binds to its receptor, it triggers signaling cascades that promote an anti-inflammatory macrophage phenotype, reducing the release of pro-inflammatory cytokines and increasing the production of anti-inflammatory cytokines like interleukin-10 (IL-10). This shift toward an anti-inflammatory profile is beneficial in reducing the overall inflammatory burden on tissues, particularly in the cardiovascular system, where inflammation is a key driver of atherosclerosis and heart disease.

Additionally, GLP-1’s effects on cytokine regulation extend to the central nervous system, where it has shown neuroprotective properties. By modulating cytokine reléase [32], GLP-1 can help reduce neuroinflammation, which is implicated in neurodegenerative diseases such as Alzheimer’s and Parkinson’s. Experimental studies suggest that GLP-1 analogs decrease levels of neurotoxic cytokines, potentially slowing down the progression of neuronal damage [33].

GLP-1 not only plays a role in glucose regulation but also significantly impacts cytokine-driven inflammation. By reducing pro-inflammatory cytokines and promoting an anti-inflammatory environment, GLP-1 and its analogs offer promising therapeutic options for managing chronic inflammatory conditions in metabolic and neurodegenerative diseases. This dual role of GLP-1 underscores its potential as a treatment strategy that targets both metabolic and inflammatory pathways.

Consequently, DPP-4 inhibitors could potentially benefit heart failure patients not only by improving glycemic control but also by directly impacting cardiovascular health.

Despite these mechanisms, DPP-4 inhibitors have produced mixed results in heart failure outcomes across clinical trials. Some DPP-4 inhibitors have been associated with an increased risk of heart failure hospitalizations, as seen in trials such as SAVOR-TIMI 53. However, other studies, such as TECOS, did not observe an elevated risk, indicating that DPP-4 inhibitors are not uniform in their cardiovascular effects. The variability across DPP-4 inhibitors highlights the need for individualized treatment strategies in heart failure patients with diabetes.

## 4. Clinical Efficacy and Safety of DPP-4 Inhibitors in Heart Failure Patients

The management of diabetes in heart failure patients is challenging due to the need to balance glucose control without exacerbating heart failure symptoms. Traditional antidiabetic medications, such as sulfonylureas and insulin, are associated with increased risks of hypoglycemia and weight gain, which are detrimental to heart failure management. DPP-4 inhibitors, with their glucose-dependent insulin secretion, low risk of hypoglycemia, and weight neutrality, provide an attractive option for these patients. Nonetheless, concerns regarding the cardiovascular safety of DPP-4 inhibitors in heart failure emerged after some agents showed adverse outcomes in cardiovascular trials.

SAVOR-TIMI 53 was a large randomized controlled trial that evaluated the cardiovascular safety of saxagliptin, a DPP-4 inhibitor, in patients with type 2 diabetes who were at high cardiovascular risk. The trial found a statistically significant increase in the risk of heart failure hospitalizations among patients treated with saxagliptin compared to placebo. This finding raised concerns about the safety of saxagliptin in heart failure patients and led to regulatory recommendations for caution when prescribing saxagliptin to this population. The SAVOR-TIMI 53 (Saxagliptin Assessment of Vascular Outcomes Recorded in Patients with Diabetes Mellitus) clinical trial was a large, multinational, randomized study that aimed to assess the cardiovascular safety of saxagliptin, a dipeptidyl peptidase-4 (DPP-4) inhibitor, in patients with type 2 diabetes mellitus who were at high risk for cardiovascular events. DPP-4 inhibitors like saxagliptin work by blocking the DPP-4 enzyme, which degrades incretin hormones such as glucagon-like peptide-1 (GLP-1) and glucose-dependent insulinotropic peptide (GIP). These incretins help manage blood glucose by increasing insulin secretion and decreasing glucagon release, especially after meals. Given that cardiovascular risks are elevated in patients with diabetes, assessing the safety of antidiabetic drugs on heart health has become a key aspect of clinical drug evaluation [34].

SAVOR-TIMI 53 was initiated in response to guidelines from the U.S. Food and Drug Administration (FDA) and the European Medicines Agency (EMA) requiring new antidiabetic drugs to demonstrate cardiovascular safety. The study included 16,492 patients from over 700 sites in 26 countries, and participants were followed for an average of 2.1 years. Eligible participants were adults with type 2 diabetes who either had a history of cardiovascular disease or multiple risk factors for cardiovascular disease. The primary endpoints of the study focused on the composite of major adverse cardiovascular events (MACEs), which included cardiovascular death, non-fatal myocardial infarction, and non-fatal ischemic stroke.

Participants were randomized in a 1:1 ratio to receive either saxagliptin (5 mg daily or 2.5 mg for those with moderate or severe renal impairment) or a placebo, in addition to their usual standard of care, which could include other glucose-lowering agents as needed to achieve glycemic control. Importantly, the study was conducted in a double-blind, placebo-controlled fashion, meaning neither the participants nor the researchers knew who received saxagliptin or placebo during the trial.

The results of SAVOR-TIMI 53, published in 2013, indicated that saxagliptin did not increase or decrease the risk of major adverse cardiovascular events compared to placebo. The incidence of the primary endpoint (MACEs) was similar between the two groups, with a hazard ratio of 1.00 (95% CI, 0.89 to 1.12; *p* < 0.001 for non-inferiority), suggesting that saxagliptin was neither better nor worse than placebo in terms of cardiovascular risk.

However, one of the most notable and unexpected findings of the trial was a statistically significant increase in the risk of heart failure hospitalization among patients treated with saxagliptin. Specifically, 3.5% of patients in the saxagliptin group were hospitalized for heart failure compared to 2.8% in the placebo group, translating to a 27% relative increase in heart failure hospitalizations (HR 1.27; 95% CI, 1.07 to 1.51; *p* = 0.007). This raised important questions about the safety of DPP-4 inhibitors in patients at risk for or with existing heart failure [35].

The SAVOR-TIMI 53 trial also looked at secondary endpoints and the safety profile of saxagliptin. There was no observed increase in other serious cardiovascular events, such as myocardial infarction or stroke, beyond the primary composite MACE outcome. However, the increased risk of heart failure hospitalization was particularly concerning given the already elevated risk of heart failure in patients with diabetes.

Other side effects reported in the saxagliptin group included an increase in hypoglycemia, particularly among patients who were also receiving insulin or sulfonylureas. However, the rates of severe hypoglycemia were not significantly different between the saxagliptin and placebo groups. Additionally, there was no statistically significant difference in rates of pancreatitis or pancreatic cancer, which had been a concern in earlier studies of incretin-based therapies.

The findings of SAVOR-TIMI 53 had considerable implications for clinical practice. The increased risk of heart failure hospitalization prompted regulatory agencies, including the FDA, to issue safety communications and update labeling for saxagliptin, cautioning against its use in patients with a history of heart failure. The study highlighted the need for careful cardiovascular risk assessment in diabetic patients prescribed DPP-4 inhibitors and raised awareness among clinicians regarding the importance of monitoring for heart failure symptoms in patients on these medications.

The trial also emphasized the heterogeneity within the DPP-4 inhibitor class. Other DPP-4 inhibitors, such as sitagliptin, have shown a neutral cardiovascular profile in separate studies like TECOS, suggesting that the heart failure risk may not be a class effect but rather specific to saxagliptin or certain patient populations. These observations underscored the necessity of conducting individual cardiovascular outcomes trials for each new diabetes medication to ensure comprehensive understanding of their safety profile in diverse patient groups.

The SAVOR-TIMI 53 findings highlighted several areas for future research. First, understanding the mechanisms behind the increased heart failure risk associated with saxagliptin could help identify specific patient subgroups at higher risk and guide more targeted treatment strategies. Research into whether certain pharmacodynamic or pharmacokinetic properties of saxagliptin contribute to this risk could shed light on underlying mechanisms.

Moreover, subsequent studies are needed to determine whether combination therapies, such as pairing DPP-4 inhibitors with other agents like SGLT2 inhibitors (which have shown favorable outcomes in heart failure), could offset the heart failure risk. Studies that examine the impact of DPP-4 inhibitors on biomarkers of heart failure and myocardial function could further refine our understanding of the relationship between DPP-4 inhibition and cardiac health [36].

The SAVOR-TIMI 53 trial was a pivotal study in cardiovascular diabetes research, affirming the cardiovascular safety of saxagliptin regarding MACEs while revealing an unexpected risk of heart failure hospitalization. The trial’s findings have influenced clinical guidelines and raised awareness about the need for cardiovascular safety monitoring in diabetes treatments. SAVOR-TIMI 53 underscored the importance of individualized patient care and the potential for nuanced differences within drug classes, setting a standard for the comprehensive cardiovascular evaluation of antidiabetic therapies.

EXAMINE, a trial evaluating the DPP-4 inhibitor alogliptin in patients with recent acute coronary syndrome, observed a neutral effect on cardiovascular outcomes, though the results suggested a non-significant trend toward increased heart failure risk. However, due to the limited sample size and short duration, the findings did not fully clarify the safety of alogliptin in heart failure. The EXAMINE trial (Examination of Cardiovascular Outcomes with Alogliptin versus Standard of Care) was a landmark cardiovascular outcomes trial designed to assess the safety of alogliptin, a dipeptidyl peptidase-4 (DPP-4) inhibitor, in patients with type 2 diabetes who had a recent history of acute coronary syndrome (ACS). The trial emerged in response to new regulatory requirements for cardiovascular outcomes studies (CVOTs) in diabetes, following concerns that some glucose-lowering therapies might increase cardiovascular risk. The EXAMINE trial specifically aimed to determine whether alogliptin increased the incidence of major adverse cardiovascular events (MACEs) in patients with high cardiovascular risk due to their recent ACS, such as myocardial infarction or unstable angina [37].

The EXAMINE trial was a randomized, double-blind, placebo-controlled study that enrolled 5380 patients with type 2 diabetes from multiple countries. Participants were required to have experienced an ACS event within the previous 15 to 90 days before enrollment, placing them at high risk for future cardiovascular events. To be eligible, patients also needed to have glycated hemoglobin (HbA1c) levels between 6.5% and 11% at baseline, underscoring the need for ongoing glycemic management [38].

Participants were randomized to receive either alogliptin or a placebo in addition to standard care, which allowed for the use of other glucose-lowering therapies as needed. The dose of alogliptin was adjusted based on renal function (25 mg daily for normal renal function, 12.5 mg for moderate renal impairment, and 6.25 mg for severe renal impairment), reflecting the drug’s primary metabolism through the kidneys. Patients were followed for a median of 18 months, and the primary outcome was the composite of cardiovascular death, non-fatal myocardial infarction, and non-fatal stroke, defined as MACEs.

The EXAMINE trial results, published in 2013, found that alogliptin was non-inferior to placebo for the primary MACE outcome. The rates of the composite primary endpoint were nearly identical in both groups, with 11.3% in the alogliptin group and 11.8% in the placebo group (HR 0.96; 95% CI, 0.85 to 1.08; *p* < 0.001 for non-inferiority). This indicated that alogliptin did not increase the risk of major cardiovascular events in patients with recent ACS, thus meeting the trial’s primary endpoint for cardiovascular safety.

In terms of secondary cardiovascular endpoints, there was no significant difference in all-cause mortality, cardiovascular death, or rates of myocardial infarction and stroke between the alogliptin and placebo groups. However, one aspect that received attention was the risk of heart failure. Although EXAMINE did not demonstrate a statistically significant increase in heart failure hospitalizations, there was a numerically higher incidence of heart failure-related events in the alogliptin group, raising some concerns given previous findings from the SAVOR-TIMI 53 trial with saxagliptin, another DPP-4 inhibitor [39].

The overall safety profile of alogliptin in EXAMINE was favorable, with no major differences between the alogliptin and placebo groups in terms of hypoglycemia, renal events, or pancreatic issues, including pancreatitis. Unlike sulfonylureas or insulin, which are associated with higher rates of hypoglycemia, DPP-4 inhibitors such as alogliptin generally exhibit a low risk of hypoglycemia, and this was observed in the EXAMINE trial as well. Additionally, weight neutrality, a common feature of DPP-4 inhibitors, was maintained, which is clinically beneficial for patients with type 2 diabetes and cardiovascular risk, as weight gain can worsen metabolic and cardiovascular profiles [40].

A notable feature of EXAMINE was its focus on a high-risk population with recent ACS. While other trials, like TECOS and SAVOR-TIMI 53, included patients with varying levels of cardiovascular risk, EXAMINE uniquely concentrated on a subgroup with acute cardiovascular instability, thereby providing valuable insights into the safety of alogliptin in a highly vulnerable cohort. The findings reinforced the conclusion that alogliptin did not elevate MACE risk, supporting its safety profile when used in conjunction with standard care in patients with recent cardiovascular events.

The results of the EXAMINE trial have had important implications for the management of type 2 diabetes, particularly for patients at high cardiovascular risk. The study confirmed that alogliptin, as a DPP-4 inhibitor, does not increase the incidence of major cardiovascular events, reassuring clinicians that the drug can be used safely in patients with diabetes and recent ACS. However, the trend towards a slight increase in heart failure events, though not statistically significant, has highlighted the importance of carefully monitoring patients with a history of heart failure or those at high risk of heart failure when prescribing DPP-4 inhibitors.

This trial added to a growing body of evidence indicating that while DPP-4 inhibitors are safe in terms of MACEs, there may be nuanced effects on heart failure outcomes that warrant further exploration. The subtle differences in heart failure outcomes across trials like EXAMINE and SAVOR-TIMI 53 suggest that certain DPP-4 inhibitors may impact heart failure risk differently, underscoring the need for a more individualized approach when choosing glucose-lowering therapies for patients with diabetes and cardiovascular disease.

The EXAMINE trial also highlighted gaps in understanding the mechanistic effects of DPP-4 inhibition on the cardiovascular system, particularly concerning heart failure. Future research could investigate the physiological basis for heart failure risks associated with DPP-4 inhibitors and explore how these medications interact with other antidiabetic agents, such as SGLT2 inhibitors, which have shown positive effects on heart failure outcomes. Additionally, long-term studies with extended follow-up periods may provide deeper insights into the effects of DPP-4 inhibitors over time in high-risk populations [41].

EXAMINE further underscored the importance of conducting CVOTs for all new antidiabetic drugs, especially for those intended for high-risk patients. As diabetes management evolves, integrating these findings into clinical practice will help ensure that treatment strategies align with each patient’s specific cardiovascular profile, maximizing therapeutic benefits while minimizing risks [42].

EXAMINE trial established alogliptin as a cardiovascularly safe option for glycemic control in patients with recent ACS, reinforcing its role in diabetes care. However, ongoing vigilance is necessary to understand fully the nuanced effects of DPP-4 inhibitors on heart failure, making EXAMINE a critical foundation for future research and clinical decision-making in the management of type 2 diabetes and cardiovascular risk.

In contrast, TECOS, a trial investigating sitagliptin, found no increase in heart failure hospitalizations or adverse cardiovascular outcomes, providing reassurance regarding the cardiovascular safety of sitagliptin in patients with diabetes, including those with heart failure. Similarly, linagliptin, another DPP-4 inhibitor, has shown a neutral cardiovascular safety profile in heart failure patients, supporting its use in this population. The TECOS (Trial Evaluating Cardiovascular Outcomes with Sitagliptin) trial was a landmark cardiovascular outcomes study designed to evaluate the cardiovascular safety of sitagliptin, a widely prescribed dipeptidyl peptidase-4 (DPP-4) inhibitor, in patients with type 2 diabetes who were at risk for cardiovascular events. Conducted in response to regulatory requirements from the U.S. Food and Drug Administration (FDA) and the European Medicines Agency (EMA) for cardiovascular outcomes trials in new diabetes medications, TECOS provided essential insights into the cardiovascular effects of sitagliptin and added to the growing body of evidence on DPP-4 inhibitors in patients with diabetes and established cardiovascular disease [43].

TECOS was a multicenter, randomized, double-blind, placebo-controlled trial that enrolled 14,671 patients with type 2 diabetes and established cardiovascular disease. Participants were eligible if they had an HbA1c level between 6.5% and 8.0% while receiving stable doses of other glucose-lowering therapies, which could include metformin, sulfonylureas, insulin, or a combination of these. This HbA1c range ensured that participants had reasonably controlled blood glucose levels and reflected real-world diabetic management in patients with high cardiovascular risk.

The study’s primary endpoint was a composite measure of major adverse cardiovascular events (MACEs), including cardiovascular death, nonfatal myocardial infarction, nonfatal stroke, and hospitalization for unstable angina. Participants were randomized in a 1:1 ratio to receive either sitagliptin (100 mg daily, or 50 mg in those with moderate renal impairment) or a placebo. The trial had a median follow-up period of approximately three years, during which the researchers monitored patients for both cardiovascular events and any adverse events associated with sitagliptin.

TECOS demonstrated that sitagliptin was non-inferior to placebo in terms of cardiovascular safety. In the sitagliptin group, 11.4% of patients experienced a primary endpoint event, compared with 11.6% in the placebo group (hazard ratio 0.98; 95% confidence interval 0.89 to 1.08; *p* < 0.001 for non-inferiority). This finding meant that sitagliptin neither increased nor decreased the risk of major cardiovascular events, confirming its cardiovascular safety in patients with type 2 diabetes and established cardiovascular disease [44].

A significant aspect of the TECOS findings was the neutral effect of sitagliptin on hospitalization for heart failure. This outcome contrasted with findings from other trials on DPP-4 inhibitors, such as the SAVOR-TIMI 53 trial with saxagliptin, which had shown an increased risk of heart failure hospitalizations. In TECOS, the rate of heart failure hospitalizations was similar in both the sitagliptin and placebo groups, with no statistically significant difference (HR 1.00; 95% CI, 0.83 to 1.20). This distinction provided valuable information, indicating that not all DPP-4 inhibitors might carry the same heart failure risk and underscoring the importance of evaluating cardiovascular effects on a drug-by-drug basis within the same class [45].

TECOS provided important data on other aspects of sitagliptin’s safety profile. Hypoglycemia rates, for example, were not significantly different between the sitagliptin and placebo groups, aligning with the known lower risk of hypoglycemia associated with DPP-4 inhibitors. This safety feature makes sitagliptin a desirable option for glycemic control, particularly for patients who may have a high risk of hypoglycemia, such as the elderly or those on concurrent insulin therapy [46].

Another point of interest was sitagliptin’s neutral effect on body weight. DPP-4 inhibitors are generally weight-neutral, making them attractive for patients with diabetes who are overweight or obese. TECOS confirmed this neutral effect, with no significant difference in body weight change between the sitagliptin and placebo groups throughout the study period. Additionally, concerns surrounding the potential for pancreatic side effects, such as pancreatitis or pancreatic cancer, were addressed, with no significant differences observed between the two groups in these outcomes.

TECOS played a crucial role in shaping clinical perspectives on DPP-4 inhibitors and provided confidence in the cardiovascular safety of sitagliptin for patients with type 2 diabetes and high cardiovascular risk. The trial confirmed that sitagliptin could be used to achieve glycemic control without increasing cardiovascular risk, which is particularly important for patients with a history of cardiovascular disease. Furthermore, the neutral effect on heart failure hospitalizations in TECOS helped distinguish sitagliptin from saxagliptin and underscored the need for individualized consideration of DPP-4 inhibitors based on each drug’s cardiovascular profile.

In clinical practice, the findings of TECOS support sitagliptin as a viable option for patients needing glucose-lowering therapy, especially when cardiovascular safety is a priority. Clinicians can consider sitagliptin for patients who have concerns about heart failure or those who need to avoid hypoglycemia, as it offers a balanced approach to glucose management with minimal risk of adverse cardiovascular outcomes [47].

The TECOS trial also highlighted areas for future research. Understanding the differences in cardiovascular effects between various DPP-4 inhibitors is an area of ongoing interest, particularly given the contrasting findings with sitagliptin in TECOS and saxagliptin in SAVOR-TIMI 53 [48]. Additional studies examining the long-term effects of sitagliptin on heart failure outcomes could further clarify its place in heart failure management for diabetic patients.

Furthermore, there is growing interest in studying combinations of DPP-4 inhibitors with other antidiabetic agents, such as SGLT2 inhibitors and GLP-1 receptor agonists, both of which have shown positive cardiovascular outcomes. Understanding how these combination therapies might provide synergistic benefits for cardiovascular protection while enhancing glycemic control could offer more comprehensive treatment options for patients with type 2 diabetes and cardiovascular disease.

The TECOS trial was a pivotal study that demonstrated the cardiovascular safety of sitagliptin in patients with type 2 diabetes and established cardiovascular disease. By showing no increase in major adverse cardiovascular events or heart failure risk, TECOS provided valuable reassurance for clinicians and patients regarding the use of sitagliptin. The trial’s findings underscored the importance of individualized drug evaluation within the DPP-4 inhibitor class, helping shape the clinical approach to diabetes management with a focus on cardiovascular safety. As research continues to explore DPP-4 inhibitors and combination therapies, TECOS remains a foundational study in understanding the cardiovascular implications of diabetes medications.

The variability in cardiovascular outcomes across DPP-4 inhibitors suggests that specific agents may differ in their effects on heart failure risk. Possible explanations for these differences include variations in pharmacodynamics, binding affinity to the DPP-4 enzyme, and off-target effects on other pathways. The choice of DPP-4 inhibitor should, therefore, consider these differences and prioritize agents with proven safety profiles, such as sitagliptin and linagliptin, in patients with heart failure.

Results are summarized in Table 1.

## 5. Comparative Analysis of DPP-4 Inhibitors and Other Antidiabetic Treatments in Heart Failure

Diabetes management in heart failure patients requires careful selection of antidiabetic medications to avoid agents that exacerbate fluid retention, weight gain, or hypoglycemia. The comparison of DPP-4 inhibitors with other antidiabetic drugs reveals distinct advantages and limitations for managing diabetes in this population.

Metformin has long been the first-line therapy for type 2 diabetes, due to its efficacy, low cost, and cardiovascular safety profile. In heart failure patients, metformin is generally safe, particularly in stable heart failure without severe renal impairment. However, metformin is contraindicated in patients with advanced kidney disease due to the risk of lactic acidosis, and caution is warranted in patients with decompensated heart failure. Despite these limitations, metformin remains an essential component of diabetes management in heart failure patients. Metformin primarily works by reducing hepatic glucose production, decreasing intestinal absorption of glucose, and improving insulin sensitivity in peripheral tissues, particularly in muscle [49]. Unlike some other antihyperglycemic agents, metformin does not cause weight gain or increase the risk of hypoglycemia, making it a preferred option for many patients with T2DM. The drug has been extensively studied and is widely used due to its efficacy, safety profile, and additional benefits that extend beyond glucose control [50].

The relationship between heart failure and diabetes is significant, as patients with diabetes are at an increased risk of developing heart failure. Conversely, individuals with heart failure often experience disturbances in glucose metabolism, which can lead to the development of diabetes. This bidirectional relationship complicates the management of both conditions, as standard heart failure treatments may interact with diabetes medications, potentially worsening glycemic control or increasing the risk of cardiovascular events [51].

The use of metformin in heart failure patients, particularly those with diabetes, has been the subject of various studies, yielding mixed results. Historically, metformin was contraindicated in patients with heart failure, especially those with reduced ejection fraction (HFrEF), due to concerns about lactic acidosis, a rare but serious side effect. However, recent evidence [52] suggests that metformin may be safe and potentially beneficial in select populations with heart failure.

One of the key concerns regarding metformin in the context of heart failure is the risk of lactic acidosis, especially in patients with compromised renal function or significant hemodynamic instability. However, large observational studies have demonstrated that metformin can be safely used in patients with heart failure, provided their renal function is monitored closely. The risk of lactic acidosis in patients taking metformin is very low in practice, particularly in those with mild to moderate heart failure. The American Diabetes Association (ADA) has updated its guidelines to reflect this evolving understanding, recommending that metformin can be considered in patients with stable heart failure, especially if they have good renal function [53,54,55,56].

Metformin offers several potential benefits for patients with heart failure. Firstly, improved glycemic control is crucial for patients with diabetes and heart failure, as maintaining appropriate blood glucose levels can minimize cardiovascular risks. Metformin effectively reduces HbA1c levels, making it a valuable option for achieving this goal. Secondly, metformin is associated with weight neutrality or modest weight loss, which is particularly beneficial for overweight patients with heart failure, as obesity can exacerbate symptoms and complicate management. Furthermore, some studies have suggested that metformin may offer cardiovascular benefits beyond glycemic control. The UK Prospective Diabetes Study (UKPDS) indicated that metformin treatment was associated with a significant reduction in the risk of cardiovascular events in overweight patients with T2DM. More recent analyses have suggested that metformin may improve outcomes in patients with heart failure, particularly in those with preserved ejection fraction (HFpEF).

In addition to these benefits, metformin has demonstrated anti-inflammatory properties, which may be advantageous in heart failure management. Chronic inflammation is recognized as a contributor to the pathophysiology of heart failure, and by reducing inflammatory markers, metformin may help improve cardiac function and outcomes. However, despite these potential benefits, several challenges remain regarding the use of metformin in heart failure patients. Clinicians must carefully evaluate renal function before initiating therapy and monitor patients regularly to avoid complications. Additionally, there are still limited data from large-scale randomized controlled trials specifically evaluating the effects of metformin on heart failure outcomes. Many studies have been observational, raising concerns about confounding factors and biases [57].

As the understanding of the relationship between diabetes and heart failure evolves, further research is necessary to clarify the role of metformin in various heart failure populations. Future studies should focus on the long-term effects of metformin on heart failure outcomes, including hospitalization rates, mortality, and quality of life. Additionally, exploring the effects of metformin in specific subgroups of heart failure patients, such as those with preserved ejection fraction or varying degrees of renal impairment, could provide valuable insights into its therapeutic potential.

Heart failure remains a significant public health concern, particularly in patients with coexisting diabetes. Metformin, traditionally used for glycemic control in T2DM, has emerged as a potential therapeutic agent in the management of heart failure. While concerns about lactic acidosis have historically limited its use in this population, evolving evidence suggests that metformin can be safely utilized in patients with heart failure, especially those with well-preserved renal function. The drug offers additional benefits, such as weight management and potential cardiovascular advantages, making it a compelling option for managing diabetes in heart failure patients. Ongoing research will be essential in defining the role of metformin in heart failure management and improving clinical outcomes for this challenging patient population.

SGLT2 inhibitors have transformed the landscape of diabetes management in heart failure due to their robust cardiovascular benefits. Empagliflozin, dapagliflozin, and canagliflozin have all demonstrated significant reductions in heart failure hospitalizations and mortality in clinical trials. SGLT2 inhibitors promote osmotic diuresis and natriuresis, reducing fluid overload and lowering blood pressure, which are beneficial effects for heart failure patients. Additionally, SGLT2 inhibitors are associated with weight loss, improved renal outcomes, and reductions in major adverse cardiovascular events, making them highly suitable for diabetes management in heart failure. Sodium-glucose cotransporter 2 (SGLT2) inhibitors have emerged as a transformative class of medications for the management of type 2 diabetes mellitus (T2DM) [58,59,60], but their benefits extend well beyond glycemic control. In recent years, SGLT2 inhibitors have gained significant attention for their cardiovascular and renal protective effects, particularly in patients with heart failure (HF). This has led to a paradigm shift in the treatment of heart failure, especially for those with preserved ejection fraction (HFpEF) and reduced ejection fraction (HFrEF).

SGLT2 inhibitors work by inhibiting the reabsorption of glucose in the proximal renal tubules, leading to increased urinary glucose excretion and, consequently, lower blood glucose levels. Beyond their primary action, SGLT2 inhibitors also promote osmotic diuresis, resulting in reduced blood volume and lower blood pressure. These hemodynamic effects, along with their ability to reduce hyperglycemia, have contributed to their favorable outcomes in patients with heart failure [61].

Recent clinical trials have demonstrated that SGLT2 inhibitors significantly reduce the risk of hospitalization for heart failure and improve cardiovascular outcomes in patients with established heart failure, regardless of their diabetes status. The EMPA-REG OUTCOME trial, which assessed empagliflozin, found that this SGLT2 inhibitor not only lowered blood glucose levels but also reduced the risk of cardiovascular death and hospitalization for heart failure in patients with T2DM and established cardiovascular disease. This study marked a pivotal moment in the understanding of SGLT2 inhibitors, leading researchers to explore their direct effects on heart failure [62].

The DAPA-HF trial, which evaluated dapagliflozin in patients with heart failure and reduced ejection fraction, provided compelling evidence supporting the use of SGLT2 inhibitors in this population. The trial enrolled patients with symptomatic HFrEF, regardless of the presence of diabetes, and found that dapagliflozin significantly reduced the risk of worsening heart failure or cardiovascular death compared to placebo. Notably, this benefit was observed across a broad spectrum of patients, including those without diabetes, indicating that SGLT2 inhibitors exert direct cardioprotective effects beyond their role in glucose metabolism [63].

The mechanisms by which SGLT2 inhibitors confer cardiovascular benefits are multifaceted. One of the key mechanisms is their effect on fluid homeostasis. By promoting diuresis and natriuresis, SGLT2 inhibitors help reduce volume overload, which is a common issue in patients with heart failure. This reduction in fluid retention can lead to improvements in symptoms such as dyspnea and edema, enhancing overall quality of life. Furthermore, the decrease in blood pressure achieved through diuresis may also contribute to the prevention of further cardiovascular events.

Another important aspect of SGLT2 inhibitors is their potential to improve myocardial metabolism and function. Studies have shown that these agents may enhance the utilization of ketone bodies as an energy source for the heart, which is particularly beneficial in the context of heart failure. The heart often struggles to utilize glucose effectively in states of reduced perfusion, and SGLT2 inhibitors may help shift the energy substrate preference from glucose to ketones, thereby improving cardiac efficiency and function.

Moreover, SGLT2 inhibitors have anti-inflammatory and antioxidative properties, which may help mitigate the detrimental effects of inflammation on the heart. Chronic inflammation is recognized as a contributing factor in the progression of heart failure, and by reducing markers of inflammation, SGLT2 inhibitors may slow the progression of the disease. This dual effect on both hemodynamics and myocardial metabolism positions SGLT2 inhibitors as a novel therapeutic option in heart failure management [64,65].

The role of SGLT2 inhibitors in patients with heart failure and preserved ejection fraction (HFpEF) is an area of active investigation. HFpEF, characterized by preserved left ventricular ejection fraction but impaired diastolic function, poses significant management challenges. Early studies, including the EMPEROR-Preserved trial, are exploring the impact of empagliflozin on clinical outcomes in this patient population. While results are still pending, there is optimism that SGLT2 inhibitors may also benefit patients with HFpEF by improving diuresis, reducing hospitalizations, and enhancing overall cardiac function.

The safety profile of SGLT2 inhibitors is another important consideration in their use for heart failure. The most common side effects include urinary tract infections and genital mycotic infections due to the increased glucose in the urine. However, serious adverse events are relatively rare, and the cardiovascular benefits often outweigh these risks. Additionally, the renal protective effects of SGLT2 inhibitors, demonstrated in trials like CREDENCE, further bolster their profile as beneficial agents in patients with concurrent diabetes and chronic kidney disease, both of which are common in heart failure patients.

Despite the promising data, several questions remain regarding the long-term effects of SGLT2 inhibitors in heart failure management. Ongoing trials will help elucidate their role in specific subgroups, including those with advanced heart failure or those requiring other pharmacologic therapies. The integration of SGLT2 inhibitors into standard heart failure treatment regimens raises the need for guidelines that incorporate these agents as part of a comprehensive approach to managing heart failure, especially in patients with comorbid conditions like diabetes.

SGLT2 inhibitors represent a significant advancement in the management of heart failure. Their ability to improve cardiovascular outcomes, reduce hospitalization rates, and provide additional benefits beyond glycemic control positions them as a cornerstone in the treatment of heart failure patients. As research continues to unfold, particularly in understanding their effects in different heart failure phenotypes, SGLT2 inhibitors may become integral to enhancing the quality of life and outcomes for patients suffering from this debilitating condition. With their multifaceted benefits, SGLT2 inhibitors not only address the underlying issues of heart failure but also pave the way for more effective, holistic management strategies in an ever-growing patient population.

GLP-1 Receptor Agonists offer potent glucose control and promote weight loss, a beneficial attribute for obese heart failure patients. Certain GLP-1 receptor agonists, such as liraglutide and semaglutide, have shown cardiovascular benefits in clinical trials. However, the impact of GLP-1 receptor agonists on heart failure outcomes remains less conclusive compared to SGLT2 inhibitors. Although GLP-1 receptor agonists may not worsen heart failure, their role in reducing heart failure hospitalizations or mortality is not yet well established. Glucagon-like peptide-1 (GLP-1) receptor agonists are a class of medications that have garnered significant attention for their role in the management of type 2 diabetes mellitus (T2DM) and their potential cardiovascular benefits, particularly in the context of heart failure (HF). As the prevalence of diabetes and heart failure continues to rise globally, understanding the implications of GLP-1 receptor agonists in heart failure management is increasingly important [66,67].

GLP-1 is an incretin hormone released from the gut in response to food intake. It plays a critical role in glucose homeostasis by stimulating insulin secretion, inhibiting glucagon release, slowing gastric emptying, and promoting satiety. These actions collectively lead to reduced blood glucose levels. The pharmacological enhancement of GLP-1 signaling through GLP-1 receptor agonists mimics these effects, resulting in improved glycemic control and weight loss. Notably, GLP-1 receptor agonists include drugs such as liraglutide, semaglutide, and dulaglutide, which have demonstrated not only glucose-lowering effects but also beneficial cardiovascular outcomes [68,69,70].

The relationship between heart failure and diabetes is complex and bidirectional. Patients with diabetes are at a higher risk of developing heart failure due to several factors, including increased prevalence of coronary artery disease, hypertension, and metabolic dysfunction. Conversely, heart failure can lead to worsening glycemic control, creating a challenging clinical scenario for management. Given this interplay, GLP-1 receptor agonists have emerged as a promising therapeutic option for patients with both diabetes and heart failure [71].

Recent clinical trials have provided compelling evidence regarding the cardiovascular benefits of GLP-1 receptor agonists. The LEADER trial, which assessed liraglutide, found a significant reduction in major adverse cardiovascular events, including cardiovascular death, in patients with T2DM at high cardiovascular risk. Additionally, the SUSTAIN-6 trial demonstrated that semaglutide not only improved glycemic control but also reduced the risk of cardiovascular events, including heart failure hospitalizations, in individuals with T2DM. These findings have sparked interest in the potential direct effects of GLP-1 receptor agonists on heart failure outcomes [72].

One of the mechanisms through which GLP-1 receptor agonists may exert their cardioprotective effects is through their impact on the myocardium. Preclinical studies have suggested that GLP-1 receptor activation can improve cardiac function by enhancing myocardial glucose uptake and utilization. This effect is particularly relevant in the context of heart failure, where the heart often relies on alternative energy substrates due to impaired glucose metabolism. By facilitating glucose utilization, GLP-1 receptor agonists may improve cardiac efficiency and reduce the workload on the heart [73].

Moreover, GLP-1 receptor agonists have been shown to have anti-inflammatory and antioxidative properties, which may play a role in their cardiovascular benefits. Chronic inflammation is a well-established contributor to the progression of heart failure, and by mitigating inflammatory processes, GLP-1 receptor agonists may help protect myocardial function and prevent further deterioration. Additionally, the weight loss associated with GLP-1 receptor agonist therapy is particularly advantageous for heart failure patients, as obesity is a significant risk factor for worsening heart failure symptoms and outcomes.

The potential effects of GLP-1 receptor agonists on heart failure extend beyond those with T2DM. Emerging evidence suggests that these agents may also benefit patients with heart failure who do not have diabetes. For instance, the recent trial known as the SOUL study evaluated the effects of semaglutide on heart failure outcomes in patients without diabetes and demonstrated promising results, indicating a reduction in heart failure hospitalizations. This finding underscores the notion that GLP-1 receptor agonists may exert direct benefits on the heart, independent of their glucose-lowering effects.

Despite the promising data surrounding GLP-1 receptor agonists, several challenges and considerations remain. The optimal timing for initiating GLP-1 receptor agonist therapy in patients with heart failure is still under investigation. Clinicians must weigh the benefits of glycemic control against potential side effects, such as gastrointestinal disturbances, which can be more pronounced in patients with advanced heart failure. Furthermore, the long-term effects of GLP-1 receptor agonists on heart failure progression and outcomes require further exploration through well-designed clinical trials.

Another important consideration is the safety profile of GLP-1 receptor agonists in the context of heart failure. While these agents are generally well-tolerated, there have been concerns about their association with acute pancreatitis and potential effects on renal function. However, the overall risk appears to be low, and recent studies have not established a clear causal relationship between GLP-1 receptor agonists and significant adverse events in heart failure patients. As with any medication, careful monitoring and individualized treatment plans are crucial for optimizing patient outcomes.

The evolving understanding of the role of GLP-1 receptor agonists in heart failure management suggests several future research directions. Ongoing trials are investigating the effects of GLP-1 receptor agonists in various heart failure populations, including those with preserved ejection fraction (HFpEF) and advanced heart failure. Additionally, studies exploring the mechanisms underlying the cardiovascular benefits of GLP-1 receptor agonists may provide insights into their direct effects on cardiac function [74].

GLP-1 receptor agonists represent a promising therapeutic option for managing heart failure, particularly in patients with coexisting diabetes. The cardiovascular benefits observed in recent clinical trials, coupled with their potential effects on myocardial metabolism and inflammation, position GLP-1 receptor agonists as a key player in the evolving landscape of heart failure management. As research continues to advance our understanding of these agents, clinicians can look forward to incorporating GLP-1 receptor agonists into comprehensive treatment strategies aimed at improving outcomes for patients with heart failure. The integration of these medications into clinical practice has the potential to enhance both glycemic control and cardiovascular health, ultimately improving the quality of life for individuals affected by heart failure [75,76,77].

Insulin remains a crucial therapy for patients with advanced diabetes who cannot achieve glycemic targets with other agents. However, insulin is associated with weight gain and a risk of hypoglycemia, which can exacerbate heart failure symptoms. Fluid retention, a common side effect of insulin therapy, further complicates its use in heart failure patients. Consequently, insulin is generally reserved for patients who fail to respond to oral or injectable antidiabetic agents.

The weight-neutral and hypoglycemia-sparing profile of DPP-4 inhibitors positions them favorably for heart failure patients compared to insulin and sulfonylureas. However, the superior benefits of SGLT2 inhibitors in reducing heart failure events highlight the need for a combination approach or prioritization of SGLT2 inhibitors in heart failure patients, with DPP-4 inhibitors considered as adjunctive therapy. In recent years, there has been growing interest in the role of long-acting insulin analogs in managing diabetes among heart failure patients. Long-acting insulins, such as insulin glargine and insulin detemir, offer more stable blood glucose control with a lower risk of hypoglycemia compared to short-acting formulations. Studies suggest that these long-acting insulins may have a more favorable cardiovascular profile, although the evidence is still emerging. For instance, the use of insulin glargine has been associated with a lower incidence of heart failure-related events in patients with diabetes, indicating that not all insulin therapies are created equal in terms of their cardiovascular effects [78].

In addition to the type of insulin used, the timing and administration route of insulin therapy can impact heart failure outcomes. For instance, initiating insulin therapy in the context of acute heart failure may require caution, as the metabolic demands on the heart can change dramatically during acute decompensation. In such scenarios, the risk of hypoglycemia is heightened, necessitating careful monitoring and potentially the use of lower doses. Furthermore, the route of administration, whether subcutaneous or intravenous, can also influence the pharmacokinetics of insulin and its effects on patients with heart failure [79].

Emerging evidence suggests that newer classes of diabetes medications, such as glucagon-like peptide-1 (GLP-1) receptor agonists and sodium-glucose cotransporter 2 (SGLT2) inhibitors, may provide additional benefits for heart failure patients. These agents not only improve glycemic control but also offer cardiovascular protection and can lead to weight loss, which is particularly beneficial in individuals with heart failure. While these medications do not replace insulin therapy, they can be used in conjunction to optimize overall treatment strategies.

The importance of lifestyle modifications cannot be overstated in the management of heart failure patients with diabetes. Weight loss, increased physical activity, and dietary changes can improve insulin sensitivity, thereby reducing the need for insulin therapy. Incorporating lifestyle interventions alongside pharmacotherapy is crucial for optimizing outcomes in this patient population. Health care providers should emphasize the significance of a holistic approach that includes education on diet, exercise, and self-monitoring of blood glucose levels [80].

Additionally, the long-term implications of insulin therapy in heart failure require further investigation. The complex interactions between insulin, cardiovascular health, and metabolic control warrant ongoing research to elucidate the best strategies for managing diabetes in heart failure patients. Clinical trials exploring the safety and efficacy of insulin therapy in this population will be essential in guiding treatment recommendations and optimizing patient care [81].

Insulin plays a vital role as a drug in the management of diabetes, but its use in patients with heart failure must be approached with caution. The interplay between insulin, glucose metabolism, and cardiovascular health highlights the need for personalized treatment strategies that consider the unique challenges faced by this patient population. While insulin therapy can effectively control blood glucose levels, clinicians must be vigilant regarding its potential adverse effects on heart failure symptoms and outcomes. Utilizing long-acting insulin analogs, incorporating newer diabetes medications, and emphasizing lifestyle modifications are essential components of a comprehensive approach to managing diabetes in heart failure patients. As research continues to evolve, a deeper understanding of the intricate relationship between insulin and heart failure will pave the way for more effective therapeutic interventions, ultimately improving the quality of life and outcomes for individuals living with these complex conditions [82,83].

Hospitalization for HF is analyzed in Table 2.

## 6. Special Populations: Older Adults, Renal Insufficiency, and Other Considerations

Special populations, including older adults and patients with renal insufficiency, require tailored approaches when managing diabetes and heart failure. These populations often present with multiple comorbidities, polypharmacy, and heightened susceptibility to adverse drug effects.

In older adults, the risk of hypoglycemia, falls, and cognitive impairment associated with certain antidiabetic agents necessitates a cautious approach. DPP-4 inhibitors are generally well tolerated in older patients due to their low hypoglycemia risk and weight neutrality, which reduces the likelihood of adverse events. Moreover, DPP-4 inhibitors are administered orally and have limited drug–drug interactions, making them a suitable option for polypharmacy common in older populations.

Patients with chronic kidney disease (CKD) frequently coexist with diabetes and heart failure, and the management of diabetes in this group is challenging due to the nephrotoxicity potential of certain antidiabetic drugs. DPP-4 inhibitors are advantageous in CKD as they do not require dose adjustments in mild to moderate renal impairment. Linagliptin, in particular, is unique among DPP-4 inhibitors in that it does not require dose adjustment at any stage of renal impairment, making it a viable option for patients with advanced kidney disease. Other DPP-4 inhibitors, such as sitagliptin, do require dose adjustments based on renal function but remain relatively safe for CKD patients. Patients with diabetes frequently experience renal impairment, which can complicate treatment regimens. The kidneys play a critical role in the elimination of many medications, including several antidiabetic agents. As renal function declines, the accumulation of drugs that are primarily excreted through the kidneys can lead to adverse effects and toxicities. Therefore, selecting appropriate antidiabetic therapies in patients with renal impairment is essential for optimizing glycemic control while minimizing potential risks [84].

DPP-4 inhibitors have distinct advantages in the management of diabetes in patients with impaired renal function. Unlike some other classes of antidiabetic medications, the pharmacokinetic profiles of DPP-4 inhibitors vary, with some agents being more suitable for patients with renal impairment. For instance, sitagliptin and linagliptin have been shown to have minimal renal excretion, which allows for their use in patients with various degrees of renal dysfunction. However, dose adjustments are necessary for other DPP-4 inhibitors, such as saxagliptin and alogliptin, depending on the degree of renal impairment [85].

The safety profile of DPP-4 inhibitors in patients with renal impairment is generally favorable. Clinical trials have demonstrated that these agents do not significantly increase the risk of hypoglycemia, which is a common concern with many other antidiabetic medications. This characteristic is particularly advantageous for patients with renal impairment, as they may already be at an increased risk of hypoglycemia due to altered metabolism and excretion of drugs. The low risk of hypoglycemia associated with DPP-4 inhibitors makes them an attractive option for this population.

Moreover, DPP-4 inhibitors have been associated with other benefits that are particularly relevant for patients with renal impairment. Research has shown that these agents may have a renoprotective effect, which is crucial for patients with diabetes, as they are at a heightened risk for developing diabetic kidney disease. Studies have indicated that DPP-4 inhibitors can reduce albuminuria, a key marker of kidney damage, and may slow the progression of kidney disease [86]. This potential benefit further supports the use of DPP-4 inhibitors in patients with renal impairment, as managing both glycemic control and renal function is vital for optimizing patient outcomes.

In addition to their efficacy in managing diabetes, DPP-4 inhibitors may also have cardiovascular benefits. Cardiovascular disease is a leading cause of morbidity and mortality in patients with diabetes, and those with renal impairment are at an even greater risk. Recent clinical trials, such as the SAVOR-TIMI 53 trial for saxagliptin and the EXAMINE trial for alogliptin, have investigated the cardiovascular safety of DPP-4 inhibitors. These studies have generally demonstrated that DPP-4 inhibitors do not increase the risk of cardiovascular events, which is an essential consideration when selecting antidiabetic therapy for patients with concurrent renal impairment and cardiovascular disease [87,88].

Despite the advantages of DPP-4 inhibitors in managing diabetes in patients with renal impairment, there are some limitations and considerations to keep in mind. First, the long-term effects of these agents on renal function are still being studied. While short-term studies have shown promise in terms of renoprotection, further research is needed to establish the durability of these effects over time. Additionally, some patients may experience gastrointestinal side effects, including nausea and abdominal discomfort, which can affect adherence to therapy.

Another consideration is the potential for drug interactions. Many patients with renal impairment are likely to be on multiple medications to manage their various comorbidities. DPP-4 inhibitors can interact with other drugs, particularly those that are metabolized by the same pathways or those that may affect renal function. Therefore, it is crucial for healthcare providers to conduct a thorough medication review when initiating DPP-4 inhibitors in patients with renal impairment to minimize the risk of adverse effects.

Patient education plays a vital role in the successful management of diabetes in individuals with renal impairment. Healthcare providers should ensure that patients understand the importance of monitoring their blood glucose levels regularly and recognizing signs of hypoglycemia. Moreover, patients should be educated about the significance of maintaining adequate hydration and adhering to prescribed medications, including any necessary dose adjustments based on changes in renal function [89,90].

As the prevalence of diabetes and renal impairment continues to rise, the demand for effective and safe antidiabetic therapies will only increase. DPP-4 inhibitors represent a valuable option for managing T2DM in patients with renal impairment due to their favorable safety profile, low risk of hypoglycemia, and potential renoprotective effects. As ongoing research continues to clarify the long-term benefits and safety of DPP-4 inhibitors in this population, healthcare providers must remain vigilant in individualizing treatment plans to optimize glycemic control while safeguarding renal function.

DPP-4 inhibitors are an important class of medications for managing diabetes in patients with renal impairment. Their unique pharmacokinetic profiles, favorable safety profile, and potential benefits on renal and cardiovascular outcomes make them a valuable therapeutic option. However, careful consideration of renal function, potential drug interactions, and patient education are essential components of effective management. By understanding the nuances of DPP-4 inhibitors and their role in treating patients with renal impairment, healthcare providers can enhance the quality of care and improve the overall health outcomes for this vulnerable population. As research continues to advance our understanding of diabetes management in the context of renal impairment, DPP-4 inhibitors will likely remain a cornerstone of therapy, providing both glycemic control and potential renal protection.

Major outcomes in renal function are summarized in Table 3.

## 7. Differences Within the DPP-4 Inhibitor Class: Saxagliptin, Alogliptin, Sitagliptin, and Linagliptin

While DPP-4 inhibitors share a common mechanism of action, clinical studies reveal differences in cardiovascular safety across individual drugs. These variations underscore the importance of selecting specific DPP-4 inhibitors in heart failure patients based on their cardiovascular risk profile.

Saxagliptin: The SAVOR-TIMI 53 trial raised concerns about saxagliptin, showing an increased risk of heart failure hospitalizations. The reason for this elevated risk is not fully understood, but it may relate to off-target effects or unique pharmacodynamic properties. Consequently, saxagliptin is often avoided in heart failure patients or prescribed with caution.

Alogliptin: The EXAMINE trial reported a neutral effect on cardiovascular outcomes for alogliptin, though a trend toward increased heart failure risk was noted in some subgroups. This has led to mixed opinions regarding its use in heart failure, with cautious application in patients who have not responded to other agents.

Sitagliptin: The TECOS trial demonstrated that sitagliptin does not increase heart failure risk, providing reassurance for its safety in heart failure patients. Sitagliptin is, therefore, considered a safer choice among DPP-4 inhibitors for patients with concurrent diabetes and heart failure.

Linagliptin: Linagliptin has shown a neutral cardiovascular safety profile, similar to sitagliptin. It does not require dose adjustment for renal function, making it an advantageous option for heart failure patients with renal impairment.

The variation in heart failure outcomes observed across DPP-4 inhibitors may be attributed to differences in enzyme-binding affinity, pharmacokinetics, and off-target effects. These findings underscore the importance of selecting specific DPP-4 inhibitors based on individual patient profiles rather than assuming uniform effects across the drug class.

The major DPP-4 inhibitors currently available include sitagliptin, saxagliptin, linagliptin, and alogliptin. Each of these agents has unique pharmacokinetic and pharmacodynamic profiles that may influence their efficacy and safety in patients with heart failure. It is essential to examine how these differences can impact clinical outcomes, particularly in the context of cardiovascular health.

Sitagliptin is primarily excreted by the kidneys, necessitating dose adjustments in patients with renal impairment. It is typically administered at 100 mg once daily, with a reduced dose of 50 mg for patients with moderate renal dysfunction and 25 mg for those with severe renal impairment.

Saxagliptin, on the other hand, is also predominantly eliminated via the kidneys. The standard dosage is 5 mg daily, which can be reduced to 2.5 mg in patients with moderate to severe renal impairment. Notably, saxagliptin has been associated with an increased risk of heart failure hospitalization, particularly in patients with existing cardiovascular disease or heart failure.

Linagliptin has a distinct advantage in this comparison as it is primarily excreted via the liver, allowing it to be used without dose adjustments in patients with renal impairment. This characteristic makes linagliptin particularly appealing for use in patients with heart failure who may also have compromised kidney function.

Alogliptin, similar to sitagliptin and saxagliptin, requires dose adjustments in patients with renal impairment. The typical dosage is 25 mg once daily, which is reduced to 12.5 mg in those with moderate renal dysfunction and to 6.25 mg for patients with severe renal impairment.

The clinical efficacy of DPP-4 inhibitors in glycemic control is relatively similar across the different agents. However, their effects on cardiovascular outcomes vary significantly.

Sitagliptin has demonstrated a neutral effect on cardiovascular outcomes in the SAVOR-TIMI 53 trial, which assessed the safety and efficacy of saxagliptin. While saxagliptin did not significantly increase cardiovascular risk, it did not provide notable cardiovascular benefits either.

Saxagliptin raised concerns in the same SAVOR-TIMI 53 trial, where it was associated with a higher rate of hospitalizations for heart failure compared to placebo. This finding has led to recommendations for caution when prescribing saxagliptin to patients with a history of heart failure or those at high risk for developing heart failure.

Linagliptin has shown a more favorable cardiovascular profile. The CARMELINA trial investigated the cardiovascular safety of linagliptin and found that it did not increase the risk of cardiovascular events, making it a safe option for patients with existing cardiovascular disease or heart failure.

Alogliptin has been evaluated in the EXAMINE trial, which assessed its cardiovascular safety. The results showed that alogliptin did not increase the risk of major adverse cardiovascular events. However, the trial did not provide conclusive evidence regarding its impact on heart failure outcomes, highlighting the need for further studies.

When evaluating DPP-4 inhibitors in the context of heart failure, it is crucial to consider their effects on heart function and related outcomes.

Sitagliptin has not been associated with significant adverse effects on heart failure, but it also does not appear to offer protective effects in this patient population. Its use in heart failure patients is generally considered safe, particularly for those who do not have significant renal impairment.

Saxagliptin’s association with an increased risk of heart failure hospitalization raises concerns, especially given that many patients with T2DM also present with underlying cardiovascular conditions. The mechanism underlying saxagliptin’s potential risk may relate to its pharmacological effects on the cardiovascular system, potentially leading to fluid retention and worsening heart failure symptoms.

Linagliptin is noteworthy for its lack of negative impact on heart failure, combined with its favorable effects on renal function. The CARMELINA study demonstrated that linagliptin not only provides effective glycemic control but may also contribute to the preservation of renal function, making it an excellent choice for diabetic patients with heart failure, particularly those with renal impairment.

Alogliptin appears to have a neutral effect on heart failure, although the data remain limited. More research is needed to ascertain its long-term effects on heart failure outcomes.

The mechanisms by which DPP-4 inhibitors exert their effects on cardiovascular and renal health are of paramount importance. DPP-4 inhibitors enhance the levels of incretin hormones, which are known to have various cardioprotective effects, such as improving endothelial function and reducing inflammation. However, the degree to which each DPP-4 inhibitor influences these mechanisms varies.

Saxagliptin’s potential to cause fluid retention and heart failure exacerbations may be attributed to its unique pharmacokinetics, which lead to greater inhibition of DPP-4 compared to other agents. In contrast, linagliptin’s favorable profile may be related to its sustained incretin effect, which does not seem to interfere with fluid balance or exacerbate heart failure.

Patient-specific factors must also be taken into account when prescribing DPP-4 inhibitors. For patients with a history of heart failure or those at risk for developing heart failure, linagliptin may be the most appropriate choice given its safety profile. In contrast, saxagliptin may be best avoided due to its association with increased hospitalizations for heart failure.

In patients with renal impairment, linagliptin remains the most favorable option, as it does not require dose adjustment and poses minimal risk for worsening kidney function. In contrast, sitagliptin, saxagliptin, and alogliptin require careful consideration of renal function when determining dosing.

While all DPP-4 inhibitors serve the primary purpose of improving glycemic control in patients with T2DM, their differing effects on cardiovascular outcomes, particularly in the context of heart failure, cannot be overlooked. Saxagliptin is associated with an increased risk of heart failure hospitalization, making it less suitable for patients with existing heart failure. Conversely, linagliptin offers a favorable profile, demonstrating cardiovascular safety and potential renal benefits, making it an excellent choice for this population. Sitagliptin and alogliptin provide neutral effects on heart failure but require careful consideration in patients with renal impairment. Understanding these differences is crucial for clinicians when making treatment decisions, ultimately aiming to optimize both glycemic control and cardiovascular health in patients with type 2 diabetes mellitus. As ongoing research continues to clarify the safety and efficacy of these agents, it will be essential for healthcare providers to remain informed and vigilant in their prescribing practices [91,92,93,94,95,96,97].

## 8. Future Lines of Study

As DPP-4 inhibitors continue to be used in patients with diabetes and heart failure, there remain important areas for further research:

1. Mechanistic Studies: Further studies are needed to elucidate the molecular mechanisms by which certain DPP-4 inhibitors influence heart failure risk. Investigating the interaction between DPP-4 inhibition, GLP-1 pathways, and myocardial function could provide insights into safer drug design.

2. Long-Term Outcomes in Special Populations: More research on the long-term safety and efficacy of DPP-4 inhibitors in older adults and patients with advanced CKD is warranted. These populations are frequently underrepresented in clinical trials, yet they represent a large proportion of patients with diabetes and heart failure.

3. Combination Therapies: Examining the combination of DPP-4 inhibitors with other cardioprotective agents, such as SGLT2 inhibitors, could reveal synergistic effects in heart failure patients. Understanding the potential benefits and risks of such combinations may allow for more effective and tailored treatments.

4. Personalized Medicine: Exploring the role of genetic factors in predicting response to DPP-4 inhibitors could lead to more personalized treatment approaches in heart failure patients, allowing for individualized selection of antidiabetic agents based on genetic predispositions.

## 9. Conclusions

DPP-4 inhibitors offer a viable option for managing diabetes in heart failure patients, given their efficacy in glycemic control and generally favorable safety profile. However, the observed differences within the DPP-4 inhibitor class, especially regarding heart failure risk, emphasize the importance of individualized treatment decisions. Sitagliptin and linagliptin demonstrate the most reassuring profiles, whereas caution is warranted with saxagliptin. In comparison to other antidiabetic therapies, DPP-4 inhibitors are preferable to agents associated with hypoglycemia and weight gain, though SGLT2 inhibitors exhibit superior benefits in heart failure [98].

Future research will continue to clarify the role of DPP-4 inhibitors in managing heart failure in diabetes, particularly as we gain insight into molecular mechanisms and long-term safety in special populations. In a landscape of complex comorbidities and evolving therapeutic options, DPP-4 inhibitors remain a valuable tool, contributing to the diverse armamentarium needed to manage diabetes in heart failure patients.

## Figures and Tables

**Table 1:** Key clinical trials involving DPP-4 inhibitors.

Trial Name	DPP-4 Inhibitor	Dose	Study Population	Duration	Key Cardiovascular Findings	Other Findings
SAVOR-TIMI 53	Saxagliptin	5 mg daily (2.5 mg if eGFR < 50 mL/min/1.73 m^2^)	16,492 patients with type 2 diabetes and high CV risk	2.1 years	No increase in primary MACEs (CV death, MI, stroke); 27% increase in hospitalization for heart failure	Improved glycemic control but increased risk of hospitalization for HF
EXAMINE	Alogliptin	25 mg daily (12.5 mg if moderate renal impairment; 6.25 mg if severe renal impairment)	5380 patients with type 2 diabetes and recent acute coronary syndrome (ACS)	1.5 years	No increase in MACEs; no significant increase in heart failure hospitalizations	No significant renal adverse effects
TECOS	Sitagliptin	100 mg daily (50 mg if eGFR < 50 mL/min/1.73 m^2^)	14,671 patients with type 2 diabetes and established CV disease	3 years	No increase in MACEs or heart failure hospitalizations	Well-tolerated with a neutral effect on CV and renal outcomes
CARMELINA	Linagliptin	5 mg daily	6979 patients with type 2 diabetes and high CV and renal risk	2.2 years	No increase in MACEs or heart failure hospitalizations	Stable renal outcomes; reduced albuminuria
CAROLINA	Linagliptin vs. Glimepiride	5 mg daily vs. glimepiride (1–4 mg daily)	6033 patients with type 2 diabetes at high CV risk	6.3 years	Linagliptin non-inferior to glimepiride in MACE outcomes	Linagliptin showed lower rates of hypoglycemia compared to glimepiride
GLORIA-AF	Various DPP-4 inhibitors	Varies by drug	56,000 patients with diabetes and atrial fibrillation (AF)	Varies by study	DPP-4 inhibitors did not significantly increase incidence of AF	Study focused on atrial fibrillation incidence

**Table 2:** Hospitalization for heart failure outcomes in major DPP-4 inhibitor trials.

Trial Name	DPP-4 Inhibitor	Heart Failure (HF) Outcome	Change in HF Hospitalizations	Interpretation
SAVOR-TIMI 53	Saxagliptin	Increased HF hospitalizations	27% increase in HF hospitalizations	Higher risk for HF hospitalization in saxagliptin group
EXAMINE	Alogliptin	No significant effect	No significant increase in HF hospitalizations	Alogliptin did not increase HF hospitalizations
TECOS	Sitagliptin	No significant effect	No increase in HF hospitalizations	Sitagliptin was neutral on HF outcomes
CARMELINA	Linagliptin	No significant effect	No increase in HF hospitalizations	Linagliptin was safe with respect to HF outcomes
CAROLINA	Linagliptin vs. Glimepiride	No significant effect	Comparable HF hospitalization rates to glimepiride	Linagliptin had no adverse HF effects compared to sulfonylurea
GLORIA-AF	Various DPP-4 inhibitors	No significant increase in AF incidence	No reported increase in HF outcomes	AF-focused study, not specifically for HF

**Table 3:** Renal outcomes in major DPP-4 inhibitor trials.

Trial Name	DPP-4 Inhibitor	Study Population	Renal Outcome	Effect on Kidney Function	Interpretation
SAVOR-TIMI 53	Saxagliptin	High CV risk, some with renal impairment	Secondary renal outcomes assessed	No significant effect on progression of renal disease	Saxagliptin had a neutral effect on kidney function over time
EXAMINE	Alogliptin	Post-ACS, some with renal impairment	Secondary renal outcomes assessed	No significant deterioration in kidney function	Alogliptin was safe in patients with renal impairment
TECOS	Sitagliptin	High CV risk, some with renal impairment	Secondary renal outcomes assessed	No significant change in eGFR or albuminuria	Sitagliptin demonstrated renal safety, with stable eGFR and albuminuria
CARMELINA	Linagliptin	High CV and renal risk	Primary and secondary renal outcomes assessed	Slowed progression of albuminuria, no worsening of eGFR	Linagliptin showed favorable effects on albuminuria progression
CAROLINA	Linagliptin vs. Glimepiride	High CV risk, some with renal impairment	Secondary renal outcomes assessed	No significant impact on eGFR; similar to glimepiride	Linagliptin was non-inferior to glimepiride for renal outcomes
GLORIA-AF	Various DPP-4 inhibitors	Patients with diabetes and atrial fibrillation	No specific renal outcomes measured	Not applicable	Study focused on atrial fibrillation, not renal function
